# Accuracy of the Verbal Autopsy questionnaire in the diagnosis of COVID-19 deaths in a Brazilian capital

**DOI:** 10.1590/S1678-9946202466033

**Published:** 2024-05-13

**Authors:** Marcos Adriano Garcia Campos, Ézio Arthur Monteiro Cutrim, Érico Murilo Monteiro Cutrim, João Victor Pimentel de Oliveira, Eduardo José Silva Gomes de Oliveira, Daniel de Brito Pontes, José Albuquerque de Figueiredo, Gyl Eanes Barros Silva

**Affiliations:** 1 Universidade Estadual Paulista, Faculdade de Medicina de Botucatu, Hospital das Clínicas, Botucatu, São Paulo, Brazil; 2 Universidade Federal do Maranhão, Faculdade de Medicina, São Luís, Maranhão, Brazil; 3 Hospital Geral Dr. Cesar Cals, Fortaleza, Ceará, Brazil; 4 Centro Universitário do Maranhão, Faculdade de Medicina, São Luís, Maranhão, Brazil; 5 Universidade de São Paulo, Faculdade de Medicina de Ribeirão Preto, Departamento de Patologia e Medicina Legal, Ribeirão Preto, São Paulo, Brazil

**Keywords:** COVID-19, Verbal Autopsy, Accuracy

## Abstract

The Verbal Autopsy (VA) is a questionnaire about the circumstances surrounding a death. It was widely used in Brazil to assist in postmortem diagnoses and investigate excess mortality during the Coronavirus Disease 2019 (COVID-19) pandemic. This study aimed to determine the accuracy of investigating acute respiratory distress syndrome (ARDS) using VA. This is a cross-sectional study with prospective data collected from January 2020 to August 2021 at the Death Verification Service of Sao Luis city, Brazil. VA was performed for suspected COVID-19 deaths, and one day of the week was randomly chosen to collect samples from patients without suspected COVID-19. Two swabs were collected after death and subjected to reverse transcription-polymerase chain reaction (RT-PCR) for SARS-CoV-2 detection. Of the 250 cases included, the VA questionnaire identified COVID-19-related ARDS in 67.2% (52.98% were positive for COVID-19). The sensitivity of the VA questionnaire was 0.53 (0.45–0.61), the specificity was 0.75 (0.64–0.84), the positive predictive value was 0.81 (0.72–0.88), and the negative predictive value was 0.44 (0.36–0.53). The VA had a lower-than-expected accuracy for detecting COVID-19 deaths; however, because it is an easily accessible and cost-effective tool, it can be combined with more accurate methods to improve its performance.

## INTRODUCTION

The declaration of severe acute respiratory syndrome coronavirus 2 (SARS-CoV-2) as a pandemic by the World Health Organization (WHO)^1^ had several effects on the functioning of the healthcare system, including the interruption of nonessential services. This interruption was associated with patients’ fear of exposure to the virus and resulted in significant changes in the dynamics of available services and hospitals, a decrease in patient flow in healthcare services, and an increase in all-cause deaths at home^2-4^.

Coronavirus Disease 2019 (COVID-19) deaths alone do not explain the increase in all-cause deaths observed in Brazil^5^, which may be attributed to underreporting of COVID-19 as a cause of death^5-7^, as well as a decrease in the use of and access to healthcare services, economic difficulties, and a reduction in social support^8-11^. Additionally, individuals who have recovered from COVID-19 may be at increased risk of mortality^12^.

Although autopsies are an important diagnostic tool for evaluating unwitnessed or unexplained deaths, according to various technical guidelines, their use was reduced or suspended during the COVID-19 pandemic due to the risk of disease transmission to the professionals involved^13,14^. Because of its low cost and its promotion by the Brazilian Ministry of Health, the Verbal Autopsy (VA) questionnaire was widely used in Brazil to assist in postmortem diagnoses and investigate the increase in all-cause mortality observed during the pandemic^15^. The VA is a questionnaire administered to family members or caregivers of the deceased, which collects information on the circumstances, signs, and symptoms of illness associated with the death of an individual^16^.

However, there are still doubts about the validity of the VA for assessing individuals suspected of having died from COVID-19. Therefore, this study aimed to determine the specificity and sensitivity of the VA in the diagnosis of COVID-19 in the investigation of deaths from acute respiratory distress syndrome (ARDS) and other unknown causes.

## MATERIALS AND METHODS

This cross-sectional study, which included prospective data collection, was conducted from January 2020 to August 2021 at the Death Verification Service (DVS) in Sao Luis city, Brazil. The DVS receives cases of unwitnessed or unexplained deaths from all over Maranhao State, which has a population of just over 7 million, an area similar in size to that of Italy, but with lower human development indices^17^.

During the study period, the DVS protocol included the administration of the VA for suspected COVID-19 deaths. Furthermore, one day per week was randomly chosen to collect data from all patients. Cases of suspected COVID-19 were those in which an individual met the clinical criteria (fever, cough, generalized weakness and fatigue, headache, myalgia, sore throat, coryza, dyspnea, nausea and diarrhea, anorexia, and ARDS) at least two weeks before death, based on data from the individual’s medical records and/or reports from family members.

Two swabs were collected from each individual (one from the nasopharynx and one from the oral cavity), rotating the swab around each collection site for 10 s 6–24 h after death. All professionals involved in the collection of data for this study received one week of training on our protocol. Reverse transcription-polymerase chain reaction (RT-PCR) was performed at the Central Laboratory of Maranhao within 72 h of collection to detect SARS-CoV-2. The administration of the VA questionnaire and the collection of specimens were both performed by an experienced pathologist. The swabs were stored separately in a vertical position in test tubes containing 3 mL of saline solution and refrigerated at 4 °C until processing. The VA questionnaire used in this study was developed by the Brazilian Ministry of Health^18,19^.

A total of 409 autopsies were performed according to the study protocol and considered eligible for inclusion, of which 74 suspected and 85 non-suspected COVID-19 cases were excluded due to errors in specimen collection or processing, leaving a final sample of 250 cases. The cause of death for each case was classified according to the result of the VA. The causes included were: ARDS; cardiovascular disease (acute myocardial infarction, stroke, pulmonary thromboembolism, cardiac tamponade, mesenteric infarction); infectious disease (non-pulmonary sepsis, tuberculosis, opportunistic infections); and others (cancer, malnutrition, hypovolemia).

Age was stratified, with a significant trend toward older adults (most of the sample). Origin was classified according to the place of death: home; hospital (admitted dead on arrival); or public place (found on the street or in a public space).

Deaths were classified as positive (+) or negative (−) for suspected COVID-19 based on the responses to the VA questionnaire and the presence of SARS-CoV-2 detected by RT-PCR as follows: true positive (TP), VA+ and RT-PCR+; false positive (FP), VA+ and RT-PCR−; false negative (FN), VA− and RT-PCR+; and true negative (TN), VA− and RT-PCR−. Sensitivity was calculated using the equation TP/(TP+FN) specificity was calculated using the equation TN/(TN+FP) , and analyses were performed using RStudio language (version 4.0.2, RStudio, Inc., Boston, MA, USA).

The study was approved by the Research Ethics Committee of the University Hospital of the Federal University of Maranhao, protocol Nº 4.101.862.

## RESULTS

Of the 250 cases included for analysis in this study, more than half were negative for COVID-19 (140/250; 56.0%), while the results of the VA questionnaire indicated that 66.8% of the cases were suspected positives (Table 1). Most patients (60.0%) died in residential areas.

**Table 1:** Clinical data of the deceased individuals with and without SARS-CoV-2 detection by RT-PCR and VA performance to detect suspected cases.

Parameter	Total (n = 250)		COVID-19 − (n = 140)		COVID-19 + (n = 110)	

	N	%	N	%	N	%
**Sex**						
Female	95	38.0	49	51.58	46	48.42
Male	155	62.0	91	58.71	64	41.29
**Origin**						
Home	150	60.0	73	48.67	77	51.33
Hospital facility	93	37.2	60	64.52	33	35.48
Public place	7	2.8	7	100	0	-
**Age (years)**						
< 30	7	2.81	7	100	0	-
30–49	42	16.87	25	59.52	17	40.48
50–69	73	29.32	42	57.53	31	42.47
> 70	127	51.0	65	51.18	62	48.82
**Cause of death**						
ARDS	168	67.2	79	47.02	89	52.98
Cardiovascular disease	37	14.8	25	67.57	12	32.43
Infectious disease	23	9.2	18	78.26	5	21.74
Cerebral disease	6	2.4	5	83.33	1	16.67
Other	16	6.4	13	81.25	3	18.75
**Suspected COVID-19 based on the VA questionnaire**						
Yes	167	66.8	78	46.71	89	53.29
No	83	33.2	62	74.69	21	25.31

SARS-CoV-19 = severe acute respiratory syndrome coronavirus 2; COVID-19 = coronavirus disease 2019; RT-PCR = reverse transcription-polymerase chain reaction; ARDS = acute respiratory distress syndrome; VA = Verbal Autopsy.

The VA questionnaire identified COVID-19-related ARDS in 67.2% of the cases evaluated (47.02% COVID-19 negative, 52.98% COVID-19 positive), of which only one was not related to a previous influenza-like illness. The true prevalence of COVID-19 in our sample was 67% (61–73%), and the sensitivity and specificity were 0.53 (0.45–0.61) and 0.75 (0.64–0.84), respectively. The calculated positive predictive value was 0.81 (0.72–0.88), while the negative predictive value was 0.44 (0.36–0.53).

## DISCUSSION

Although conventional autopsy remains the primary tool even in pandemic and humanitarian emergencies, the VA questionnaire is a simple and cost-effective tool for assessing cases with undetermined causes of death, especially in situations in which autopsies are not routinely performed^20,21^.

Most studies evaluating the VA questionnaire, however, have not been validated against the gold standard (conventional diagnostic autopsy), which typically involves medical record interpretation^22^. Therefore, we aimed to define the sensitivity and specificity of the VA questionnaire in situations in which conventional autopsies cannot be performed due to biosafety issues or excess deaths at home without medical assistance^23^.

For this study, the performance of RT-PCR assays for the diagnosis of SARS-CoV-2 in nasopharyngeal swab samples was used as the standard of comparison. Although the limitations of the detection window and variability in adequate collection can affect the results, a previous Brazilian study that used the VA questionnaire to investigate excess deaths at home in a large city showed that the rate of SARS-CoV-2 positivity among suspected cases reached 100.0% at the peak of the pandemic^24^. In other words, the VA questionnaire could be effectively used in the context of the COVID-19 pandemic with satisfactory results.

Interestingly, the results of this study showed that although the VA questionnaire had a moderate specificity, its performance may not be good, as the sensitivity was low. This discrepancy stems from cases in which COVID-19 was not suspected but the RT-PCR results were positive, as seen mainly in deaths from cardiovascular diseases (32.43% were COVID-19 positive). What may initially seem to be an excess of non-suspected COVID-19 deaths resulted only from the difficulty of individuals to access health services^25^, and, after obtaining the RT-PCR results, we identified COVID-19-related deaths with atypical/asymptomatic clinical presentations, lacking criteria for suspicion. This suggests that the patient died of a cause other than COVID-19 infection, although the infection was detected. Thus, the use of the VA questionnaire alone may lead to underreporting of cases. In view of these results, the isolated use of the VA questionnaire does not appear to be advantageous, contrary to what has been reported in the relevant literature.

An official data comparison study conducted in Somalia in 2020 found that 176 out of 530 (33.2%) cases were probable COVID-19 deaths, as determined using the VA questionnaire, mostly deaths at home^26^. The positive predictive value of the VA was lower for home deaths (22.3%; 95% confidence interval [CI], 15.7–30.1%) than for hospital deaths (32.3%; 95% CI, 16.7–51.4%), while the negative predictive value was higher for both home (97.8%; 95% CI, 95.0–99.3%) and hospital (98.4%; 95% CI, 91.5–100%) deaths. Furthermore, using the VA, the authors found that a higher prevalence of COVID-19 was correlated with a higher positive predictive value for both home and hospital deaths, although this value remained better for hospital deaths.

Most studies, however, have reported favorable outcomes for the VA, albeit based on poor quality and low robustness comparisons of data on cause of death, which affects the questionnaire validation^27^. Modifications have therefore been made to improve the accuracy of the VA, such as the creation of a diagnostic stratification system to categorize cases as unlikely, possible, probable, and definite^28^, and a version of the VA that uses a community-based mechanism to identify suspected COVID-19-related deaths at the population level^29^.

Another strategy is the performance of minimally invasive autopsies (MIAs), which involve the systematic collection of tissue samples from various organs and body fluids and are simple, have easy applicability, and can provide robust data for death assessment in regions with limited resources^30^. During the 2018 Brazilian yellow fever epidemic, ultrasound-guided MIAs were found to be an effective alternative to traditional autopsies^31^. MIAs have also been used to evaluate deaths caused by COVID-19^32,33^. These changes are indicative of the adaptability that autopsy protocols must hold to maintain their fundamental contributions to health surveillance systems during periods of widespread disease^34^. This is particularly true during pandemics, as death investigations using alternative autopsy practices can provide valuable data on the clinical presentation of several diseases, especially in cases of deaths at home in individuals without prior medical records^35^.

Automated algorithms can also be used to determine the cause of deaths at home or of patients whose medical records are limited or unreliable. For example, the SmartVA software for computerized VA certification uses interview results as input data from which it outputs estimated causes of death at the individual and population levels^36^. In a Brazilian study of 3,139 deceased individuals, SmartVA showed acceptable accuracy in predicting death compared to conventional autopsy for cardiovascular disease (46.8 vs. 54.0%), cancer (10.6 vs. 11.4%), infection (7.0 vs. 10.4%), and chronic respiratory disease (4.1 vs. 3.7%)^37^. However, this scenario also implies that the VA method has an important limitation: the absence of auxiliary examinations and the reliance on oral reports from families to collect data, which may underestimate some symptoms and comorbidities. In addition, VA respondents may experience emotional distress due to the interviews^38^.

## CONCLUSION

The results of this study showed that the use of the VA questionnaire alone had a lower-than-expected accuracy for detecting COVID-19-related deaths. Therefore, the use of other available tools as a supplement to detect suspected cases or individuals who died of other causes but had COVID-19 infection may be the best strategy to ensure optimal surveillance during an ongoing pandemic.
